# Deep learning application engine (DLAE): Development and integration of deep learning algorithms in medical imaging

**DOI:** 10.1016/j.softx.2019.100347

**Published:** 2019-10-29

**Authors:** Jeremiah W. Sanders, Justin R. Fletcher, Steven J. Frank, Ho-Ling Liu, Jason M. Johnson, Zijian Zhou, Henry Szu-Meng Chen, Aradhana M. Venkatesan, Rajat J. Kudchadker, Mark D. Pagel, Jingfei Ma

**Affiliations:** a Department of Imaging Physics, The University of Texas MD Anderson Cancer Center, 1515 Holcombe Blvd., Unit 1472, Houston, TX 77030, United States of America; b Medical Physics Graduate Program, MD Anderson Cancer Center UTHealth Graduate School of Biomedical Sciences, Houston, 1515 Holcombe Blvd., Unit 1472, TX 77030, United States of America; c Odyssey Systems Consulting, LLC, 550 Lipoa Parkway, Kihei, Maui, HI, United States of America; d Department of Radiation Oncology, The University of Texas MD Anderson Cancer Center, 1515 Holcombe Blvd., Unit 1422, Houston, TX 77030, United States of America; e Department of Diagnostic Radiology, The University of Texas MD Anderson Cancer Center, 1515 Holcombe Blvd., Unit 1473, Houston, TX 77030, United States of America; f Department of Radiation Physics, The University of Texas MD Anderson Cancer Center, 1515 Holcombe Blvd., Unit 1420, Houston, TX 77030, United States of America; g Department of Cancer Systems Imaging, The University of Texas MD Anderson Cancer Center, 1515 Holcombe Blvd., Unit 1907, Houston, TX 77030, United States of America

**Keywords:** Medical imaging, Software, Deep learning, Algorithm development

## Abstract

Herein we introduce a deep learning (DL) application engine (DLAE) system concept, present potential uses of it, and describe pathways for its integration in clinical workflows. An open-source software application was developed to provide a code-free approach to DL for medical imaging applications. DLAE supports several DL techniques used in medical imaging, including convolutional neural networks, fully convolutional networks, generative adversarial networks, and bounding box detectors. Several example applications using clinical images were developed and tested to demonstrate the capabilities of DLAE. Additionally, a model deployment example was demonstrated in which DLAE was used to integrate two trained models into a commercial clinical software package.

## 1. Introduction and background

Deep learning (DL) [1] techniques automatically develop parameterized algorithms, known as models, to infer useful information given sufficient data. Enabled by the confluence of large data sets, general-purpose graphics processing units (GPUs), and open-source software frameworks, modern DL techniques routinely achieve state-of-the-art human-quality performance in information-processing tasks and have demonstrated success in the domain of computer vision for medical imaging.

The general approach to developing supervised DL models for medical imaging applications is to identify a computational task, curate a data set containing images (inputs, X) and annotations (outputs, y), construct a model f(·) conforming to the input and output data domains, and train the model to accurately map the inputs to the outputs (y = f[X]) with the goal of making the trained model able to make accurate predictions on new images that it has not observed. In 2012, researchers trained a convolutional neural network (CNN) to accurately classify images, thereby establishing a new state of the art for that application domain [2]. This demonstration of capability inspired the application of DL to numerous new application domains, including medical imaging, where DL techniques have become the stateof-the-art for many tasks [3]. In the following years, public visual recognition competitions yielded refinement and extension of CNNs [4–14]. During this period, development of new model architectures resulted in substantial performance gains. The design and evaluation of model architectures to fit novel application domains remain core areas of activity for DL practitioners. Therefore, exploring a variety of architectures for a given DL application is critical to ensuring superior performance for production-level model deployment. This is especially true in the field of medical imaging, in which physicians use model predictions on patient medical images to inform their medical decisions and treatments on humans.

Researchers have proposed the use of several DL architectures, largely based on CNNs and fully convolutional networks (FCNs) [15], for medical imaging applications. Some have proposed architectures based on CNNs for medical image classification and regression tasks [16–21]. Others have developed architectures based on FCNs for medical image segmentation [22–30] and image synthesis via regression [31–38]. Additionally, some have reposed the task of object detection as a segmentation task using FCNs [39]. Arguably the most popular of these architectures—the DL workhorse in medical imaging—is UNet (and its numerous minivariants), which includes a convolutional encoder–decoder constructed with (or, to generalize, without) skip connections between the encoder and decoder at equivalent resolution scales [40,41]. Although UNet and its numerous minivariants have been successful in several medical imaging applications, it is limited to pixel-wise classification and regression tasks, which represent a small subset of the potential applications in medical imaging. Furthermore, the replication and minute variations among many UNet- and FCN-based architectures have led to repeated efforts within the DL community.

Generative adversarial networks (GANs) represent another class of DL techniques that perform information-processing tasks in medical imaging. GANs consist of two neural networks—a generator and a discriminator—that are trained to compete against one another in a minimax game [42]. GANs have demonstrated the ability to perform several tasks in medical imaging, including image synthesis [43–45], segmentation [46–48], reconstruction [49–51], and superresolution [52,53]. GANs’ ability to learn their own loss function makes them unique among all DL techniques and enables them to produce realistic samples from the data domain without explicitly programming a loss function. Because the annotations in medical images are most often paired pixel-wise with the input images, certain GANs are well suited for medical imaging applications [54]. Instances also arise in which the input images are not paired with their annotations. For example, in multiacquisition MRI protocols in which the patient may move between scans with different pulse sequences, the information content in the different images is similar, but this information is not paired pixel-wise due to patient motion. Certain GANs have demonstrated the ability to translate information from the input to the output under similar scenarios [55] and could be useful in medical imaging applications. Because FCNs and some GANs can make pixel-wise predictions on images, it is often unclear a priori which would produce superior performance for a given application. Therefore, the ability to easily and rapidly explore and compare these two approaches for a given application would be beneficial.

Object-detection tasks are common in medical image processing. Computer vision researchers have created several approaches to object detection with DL whereby FCNs are trained to predict bounding boxes (bounding box detectors [BBDs]) around multiple classes of objects within an image [56–59]. Building on these developments, researchers have proposed using BBDs in some medical imaging applications [60,61]. The general approach with BBDs is to map input images to a set of bounding box coordinates, the object class in the bounding box, and the associated predictive confidence. The training and inference procedures for BBDs require unique programmatic routines that are distinct from those used for CNNs, FCNs, and GANs. Because other DL techniques can be reposed as detection tasks, BBDs add an additional layer of complexity to selecting the appropriate DL technique for a given medical imaging application.

Given the variety of DL approaches available for novel medical imaging applications, the need for rapid prototyping of DL models in medical imaging tasks has become apparent. Moreover, many clinical workflows involving medical images inherently produce the annotations required to train DL models (e.g., segmentation masks, registered multimodal images, object/disease presence and/or location). These data sets enable new DL applications to support research programs, streamline clinical workflows, help manage treatments, and support clinical quality assurance programs. However, the design and implementation of DL models require extensive and specialized programming and computational knowledge. This creates a barrier to the application of DL techniques to clinically relevant problems by physicians and scientists. Additionally, fruitful integration of trained models into clinical workflows requires that models be encapsulated and deployed as software systems. This requirement poses even greater technical hurdles for DL adoption.

In this work, we ease DL model design, implementation, training, and deployment using a graphical user interface (GUI). We support a comprehensive set of DL architectures and loss functions for several tasks in medical imaging, and enable full automation of DL model development and inference. As such, with an appropriately curated data set, users would be able to explore a variety of DL techniques for numerous applications and integrate them into workflows using a single coherent software framework. The deep learning application engine (DLAE) is a software framework and application that enables users to design, train, validate, and coherently encapsulate and deploy a DL model while hiding programmatic implementation details. We provide a high-level overview of the design of DLAE, demonstrate separability between graphical model construction and graph processes through the use of configuration files, demonstrate the automation of training and inference processes, and present some example DL applications in medical imaging produced by DLAE.

## 2. Software framework

### 2.1. Software concept

DLAE is an application that abstracts the task of DL model development to a series of button clicks, making use of lower level libraries. Primarily, it is an application built around the Keras library [62], which is above the TensorFlow [63] and CUDA libraries.

Several prebuilt applications are included in DLAE that are compatible with many tasks encountered in medical imaging, and they can be loaded to be modified and/or trained. As such, no explicit programming is required to utilize these applucations. This is facilitated by graphical user interface input (GUI) controls which construct engine configurations (or configuration files) that store all information about a model developed for a given application. Sharing of configuration files facilitates collaborations across research groups and serves as an easy means for reproducing research results.

The engine in DLAE runs solely with configuration structures as inputs, which can be loaded from configuration files. Configuration files decouple the GUI from the processing engine. Therefore, the engine can be run without user input. Once a DL model with a specific layer configuration is developed, the configuration file defining the model can be passed to the engine to perform an automated training session. This enables streamlined ablation studies and hyperparameter searches for a given DL application development. Configuration files also facilitate the incorporation of trained DL models into workflows by enabling automated inference.

### 2.2. Software GUI

**Fig. 1.**
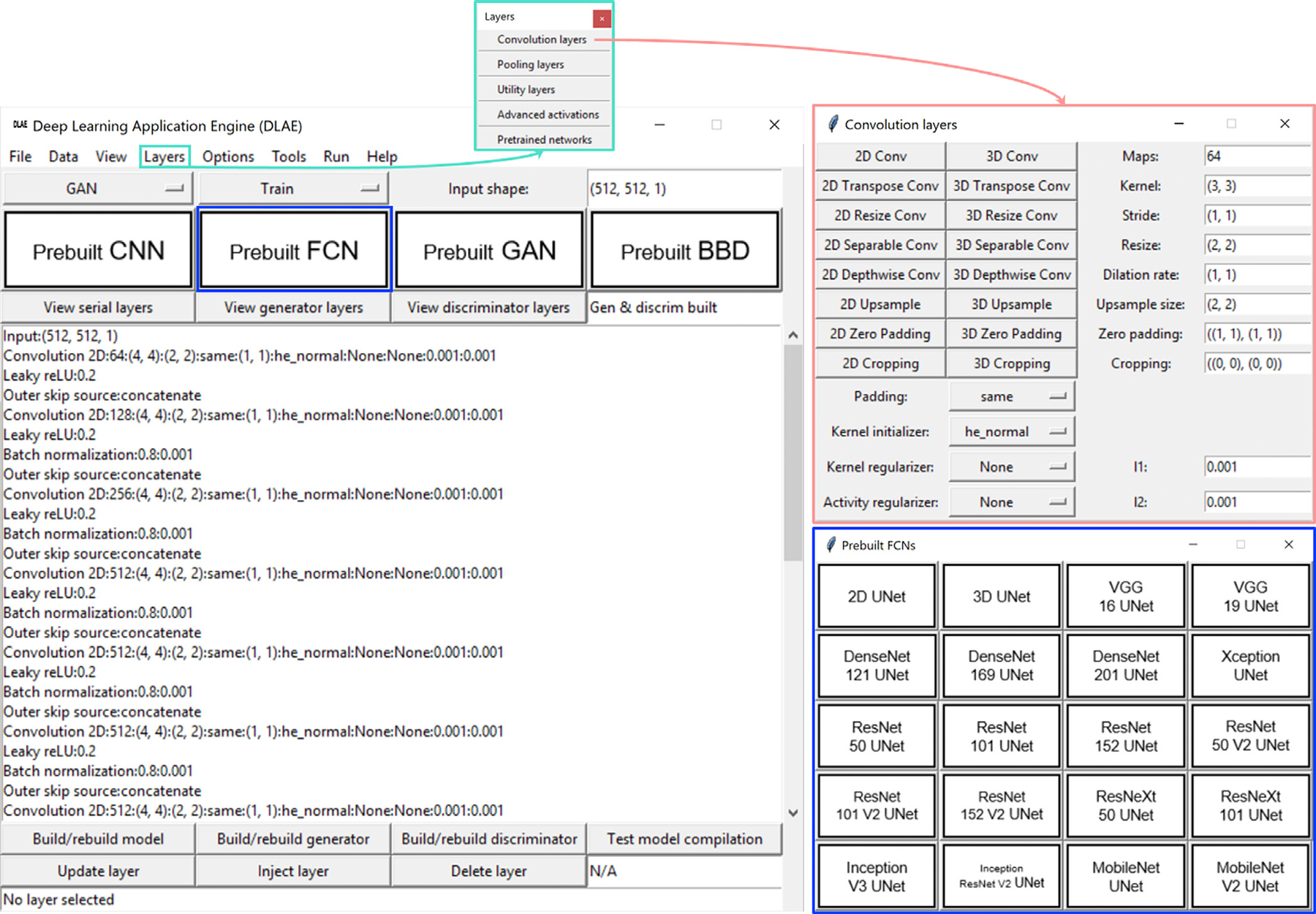
The DLAE GUI home screen was partitioned into menus, each corresponding to a step in a DL workflow. DLAE configuration files (JSON format) can be loaded or saved from the File menu. Training, validation, and testing images and annotations can be loaded using an interface in the Data menu. Options for data augmentation, and preprocessing of the images, annotations, and predictions were also included in the Data menu. The View menu allows users to view the states of the GUI parameters. The Layers menu provides the majority of the core functions for building DL models and applying transfer learning from networks pretrained on ImageNet. The Layers menu and Convolutional Layers submenu are shown as example in turquoise and red, respectively. The Options menu contains the GUI objects for specifying the loss function for training, choosing the optimizer, defining the training configurations, specifying metrics to monitor during the training process, and specifying the types of information to save from a training session. The Tools menu enables deleting a model to start over or open the most recently saved TensorBoard log in the user’s default web browser. The Run menu contains the command to run the engine based on the current GUI state. The Help menu displays error messages and can take the user to the repository where DLAE is hosted (https://github.com/jeremiahws/dlae). In addition to hosting the submenu, the DLAE GUI home screen provides a simple means of loading a number of architectures from the literature or building a custom model de novo. The prebuilt FCN submenu is shown in blue as an example. Because the GUI and engine are decoupled, the GUI serves as an interface for configuration file creation combined with an engine launch automation capability. If the user passes a configuration file to DLAE from the command line instead of running the dlae.py script, the GUI is suppressed, and either a training or inference session will be launched depending on the specifications in the configuration file.

A GUI was developed to facilitate DL model development (Fig. 1). This enables the adoption of deep neural networks without the programming experience required by modern DL frameworks. This constitutes a tradeoff, reducing the expressiveness available to DLAE users while increasing the number of individuals capable of applying modern DL models to problems in medical imaging. Knowledge of the statistical foundations underlying modern DL is still required to effectively develop applications. However, this knowledge is more common among domain experts than is the corresponding level of software engineering expertise required to field DL applications.

The GUI home screen is partitioned into menus, each corresponding to a step in a DL workflow. DLAE configuration files (JSON format) can be loaded or saved from the File menu. Training, validation, and testing images and annotations can be loaded using an interface in the Data menu. The format of the datasets for the various DL techniques are described in Section 2.3. The View menu allows users to view all of the active parameter states of the GUI. The Layers and Options menu provide a majority of the core functions for building and training DL models, respectively. They contain the GUI objects for building the layer configurations, specifying the loss function for training, applying transfer learning from networks pre-trained on ImageNet, choosing the optimizer, defining the training configurations, specifying metrics to monitor during the training process, and specifying what type of information to save from a training session. The Tools menu contains the functions for deleting a model (or generator and discriminator) to start over, or open the most recently saved TensorBoard log in the user’s default web browser. The Run menu contains the command to run the engine based on the current GUI state. The Help menu takes the user to the repository where DLAE is hosted, as well displays any errors that may be encountered during engine execution. Finally, in addition to hosting the submenu, the GUI home screen provides a simple means to load in a number of prebuilt applications, or start to build a custom model from scratch.

### 2.3. Input data formats

Input images and annotation pairs are loaded into DLAE with a consistent format. For planar (two-dimensional) images, the input format is (*n*_imgs_, *n*_rows_, *n*_cols_, *n*_chans_), in which *n*_imgs_ is the number of images in the data set, *n*_rows_ is the number of rows in the images, *n*_cols_ is the number of columns in the images, and *n*_chans_ is the number of image channels. For volumetric (three-dimensional) images, the input format is (*n*_imgs_, *n*_rows_, *n*_cols_, *n*_slices_, *n*_chans_), in which *n*_slices_ is the number of slices in the image volumes and the other parameters are the same as those for planar images.

The annotation format is specific to the type of DL model developed. For classification tasks with CNNs, the annotations are a vector of size (*n*_imgs_,), with integers in sequentially increasing order defining the object class in each corresponding image. These classes can be converted to categorical data in DLAE if desired. For regression tasks with CNNs, the annotations have the format (*n*_imgs_, *n*coords), in which *n*coords is the number of coordinates to fit in the regression. The annotations for FCNs and GANs have the same shape as the input images, allowing for pixel-wise mapping from input images to output predictions. Finally, the annotations for BBDs have a stricter requirement; they are a vector of size (*n*_imgs_,), with each entry containing an array of size (*n*_box,i_, 5) box coordinates and object classes contained within the image, where *n*_box,i_ is the number of ground truth boxes in image i *ϵ* [1, *n*_imgs_].

The images and annotations should be saved as a pair of HDF5 files. Specifically, the images should be saved as a single data set in one file, and the corresponding annotations should be saved as a single data set in the other. The exception to this format is the pair of images and annotations for BBDs. Both the images should be saved as separate data sets with the same data set name in each file. Example datasets can be found at https://github.com/jeremiahws/dlae/tree/master/datasets to help users understand the data shapes and structures.

### 2.4. Prebuilt DL models

A variety of DL architectures have been developed that are compatible with medical imaging applications. Users may either create a custom application with a user-defined layer configuration via GUI controls or load a prebuilt application with a layer configuration reported in the literature. Prebuilt CNN applications currently incorporated are: LeNet5 [64], AlexNet [2] and other CNNs that were developed on the ImageNet dataset [4–14]. FCN-based models conforming to the UNet formalism (an encoder and decoder that both use convolutions with or without skip connections between the encoder and decoder) [40,41] are also prebuilt for the user. These include FCNs that use one of the CNNs developed for image classification on ImageNet as the convolutional encoder, with a series of transpose or resize convolutions [65] as the decoders to upsample the encoding back to the input image size. GANs in the literature that are currently incorporated in DLAE include paired image-to-image translation conditional GANs (pix2pix) [54] and unpaired image-to-image cycle-consistent GANs (cycleGAN) [55]. Users can either load the original architectures reported in the literature or build their own custom generators and discriminators using GUI controls. Finally, single-shot detectors (SSDs) are currently incorporated as the BBDs in DLAE [59]. Users can load a prebuilt SSD architecture reported in the literature or build new encoders with custom predictor layers at different resolution scales via hook layers.

### 2.5. Building custom DL models

Custom DL models can be built using DLAE. All DL models, both custom and prebuilt, start with definition of the input image shape. Serial layer configurations can then be built upon the input using GUI controls. Both skip connections (for FCNs) and hook layer connections (for BBDs) can be injected at the desired resolution scales. GANs have two neural networks competing against each other, and users can specify separate custom layer configurations for the generator and discriminator. In addition, users can define a custom set of training options, learning rate schedules, and the desired loss function and optimizer to train the layer configuration for a given application.

### 2.6. Transfer learning

DLAE primarily makes use of convolutional encoders pretrained on the ImageNet data set for transfer learning. Users can employ these pretrained convolutional encoders to train a new image classifier, an FCN for semantic segmentation or image regression, or a new SSD. We added skip connections to the pretrained convolutional encoders, which give users the option of concatenating features from the encoder with the decoder at equivalent resolution scales when creating a custom FCN (i.e., in a UNet fashion). We also added hook connections to the pretrained convolutional encoders, which give users the ability to apply the pretrained convolutional encoders to new SSD applications.

### 2.7. Objective functions for training DL models

The parameters in DL algorithms are trained through optimization of some loss function. Thus, appropriate selection of the loss function is one of the most important aspects of successfully training a DL model. The loss function used to train a deep neural network depends on the type of inference task and may be application-specific. DLAE currently has seven loss functions that can be supplemented in the future:

1. Cross-entropy: for image classification with CNNs and pixel-wise classification with FCNs.
2. Mean squared error: for image regression with CNNs and pixel-wise regression with FCNs.
3. Mean absolute error: for image regression with CNNs and pixel-wise regression with FCNs.
4. Tversky index: for image segmentation with FCNs [66].
5. Conditional GAN + L1 loss: for paired image-to-image translation with conditional adversarial networks [54].
6. Adversarial + cycle consistency loss: for unpaired image-to-image translation with cycle-consistent adversarial networks [55].
7. SSD loss: for object detection with SSDs [59].
8. Jaccard distance: for image segmentation with FCNs.
9. Focal loss [67]: for image segmentation with FCNs.
10. Soft dice: for image segmentation with FCNs.

### 2.8. Additional features of DLAE

Other useful features are incorporated in DLAE. First, users can save TensorBoard summaries during training. Second, users can save model checkpoints at a specified frequency, data from monitors of the training history, and the final trained model. Third, options are available to perform data preprocessing and augmentation. Users can either perform these preprocessing steps before or have DLAE perform them. Finally, multiple GPU training is possible if they are available on the computer.

## 3. Configuration files

**Fig. 2.**
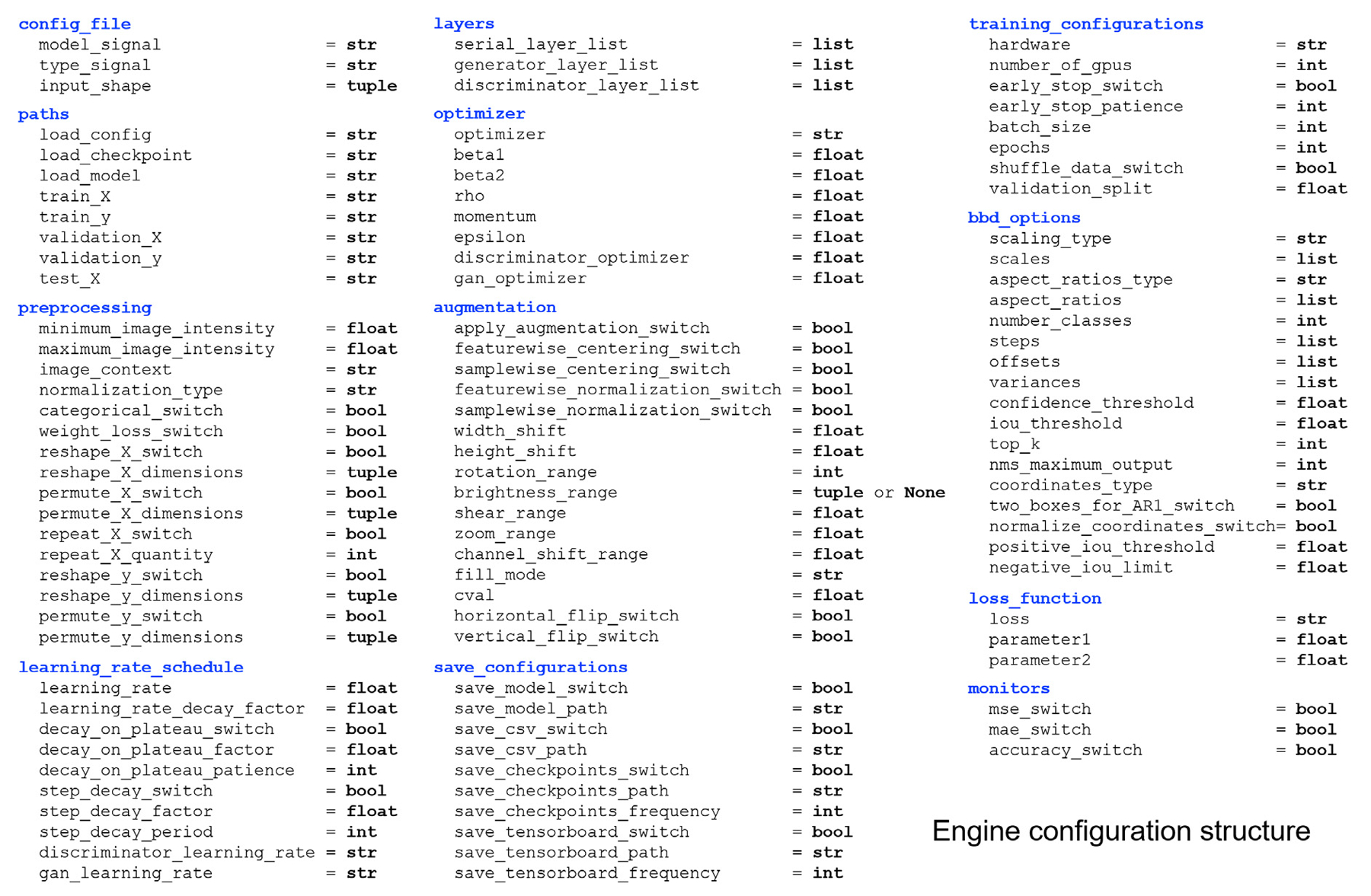
Structure of a DLAE configuration file and the data types of the variables.

Once a DL model has been created, it can be saved to a configuration file and shared by project collaborators. Given a configuration file, DLAE allows a user to view, modify, retrain, and/or make predictions using a DL model created by a collaborator. Configuration files contain all of the specifications that the engine analyzes. They include the layer configurations for a model, training specifications (batch size, epochs, etc.), which optimizer to use, learning rate schedules, the desired loss function, and all other engine configurations. The structure of a configuration file is shown in Fig. 2.

**Fig. 3.**
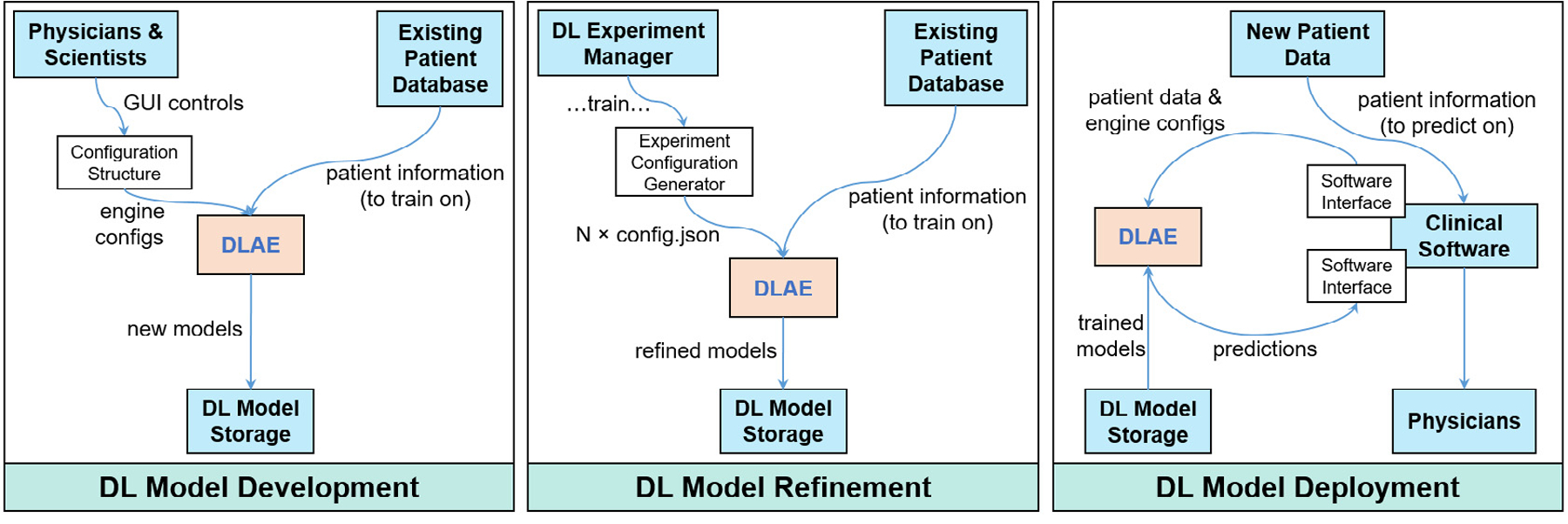
Current DL functionalities of DLAE. From left to right: (1) custom DL models can be built and trained from scratch or with warm starts via a transfer learning approach through inputs to the GUI. (2) Automated DL model training sessions can be conducted with the use of an experiment generator, which spawns DL models to be trained for a given application. This could be useful in determining the optimal DL architecture, performing ablation studies, or determining the appropriate set of hyperparameters for a given DL application. (3) Automated inference sessions can be conducted, enabling the integration of trained DL models into clinical workflows.

Configuration files decouple the GUI from the engine, which enables three primary functionalities (Fig. 3). First, after a custom model is built using the GUI and a training session is initialized, a configuration structure is passed to the engine to be executed (Section 4).

Second, configuration files enable the instantiation of several automated training sessions using an experiment generator. This is particularly useful for exploring several different DL model constructs, whereby an experiment generator can spawn several model experiments to identify the superior model for a given application. For example, users can modify layers in the configuration file of a template model to determine how certain layers impact the performance of the model for a given application, such as in ablation studies. This functionality is also useful for hyperparameter searches. For example, users can create a series of configuration files that iterate over a set of hyperparameters for a given layer configuration and pass them to the engine for automatic processing.

Third, configuration files enable automated inference, which facilitates the deployment of a trained DL model. When a configuration file is passed to the engine in inference mode, the engine performs the specified image-preprocessing steps, loads a trained model from a specified file path, and makes predictions on the images with the specified model. The inferences are stored in a file location defined in the configuration file.

## 4. Processing engine

**Fig. 4.**
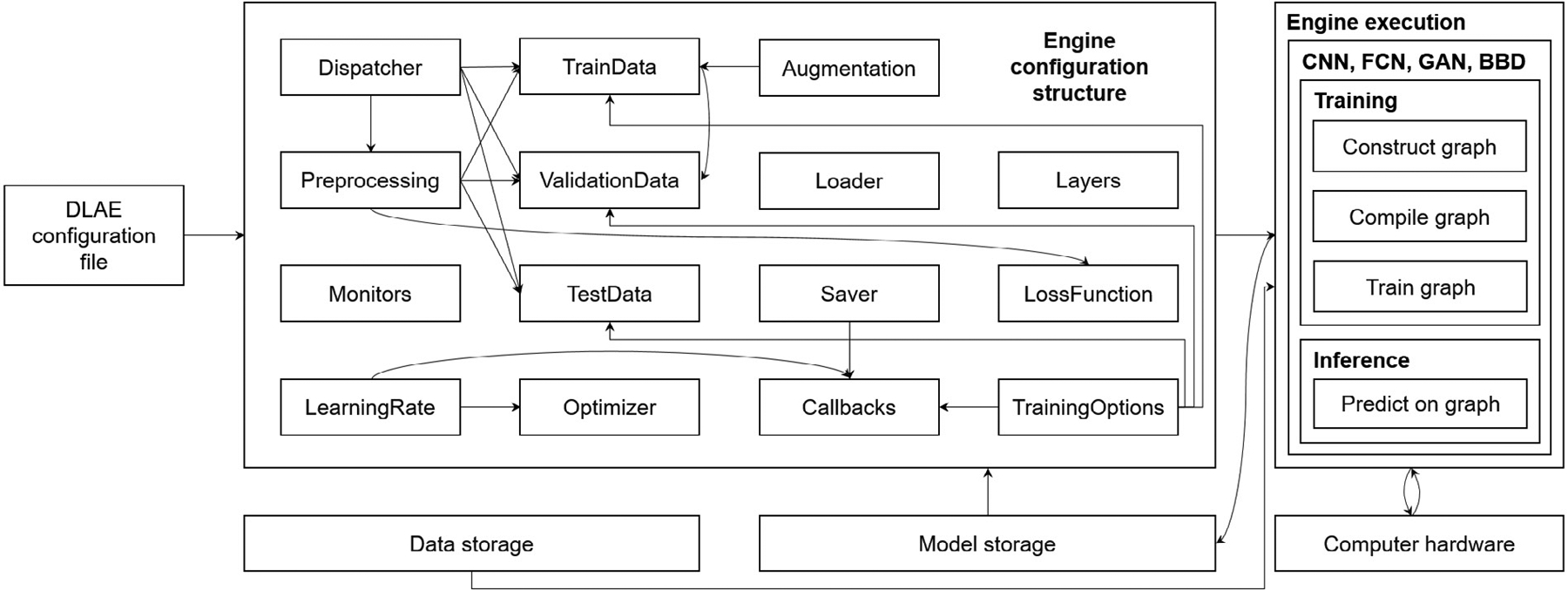
Brief overview of DLAE configuration structures and engine execution. The Dispatcher class is constructed to send signals related to the DL action to be applied. The dispatcher sends a train signal or an inference signal to the engine. A train signal instructs the engine to construct and train a DL model based on the layer configurations, objective function, and training options specified. An inference signal instructs the engine to load a trained DL model and images with which to make predictions and perform the predictions on the images. The images and annotations are loaded and prepared based on the attributes of the TrainData, ValidationData, and/or TestData classes. The data are then preprocessed based on the specifications in the Preprocessing class. If augmentation is applied for training, the training data is prepared based on the specifications in the Augmentation class. The dispatcher also sends a signal to the engine specifying the DL technique to be applied. The DL techniques currently incorporated in DLAE are CNNs, FCNs, GANs, and BBDs. For training CNNs and FCNs, the computational graph is constructed from the serial list of layers defined in the Layers class. Any inner skip connections (e.g., residual connections) or outer skip connections (e.g., concatenations between the encoder and decoder) are connected in the graph construction, and they must be specified when building the model. For training GANs, two separate networks are defined in the Layers class for the generator and discriminator. The graph construction for both the generator and discriminator follows the same construction algorithm as the serial model for CNNs and FCNs. For training BBDs, an FCN is constructed from the serial list of layers defined in the Layers class. The user must specify hook layers in the serial layers list at desired prediction resolution scales. After the graph is constructed, it is compiled with the configurations defined in the TrainingOptions, Optimizer, LearningRate, LossFunction, and Monitors classes. Multiple models are compiled for GANs: one each for the generator, discriminator, and combined generator and discriminator model. Finally, after the graph is compiled, it is trained based on the parameters defined in the TrainingOptions, Callbacks, TrainData, and ValidationData classes. If the engine receives an inference signal from the dispatcher, the engine loads a model via the Loader class and makes predictions on the data from the TestData class.

The engine in DLAE is run solely from engine configurations that can either be passed as structures from the GUI to the engine or as configuration files to the engine as inputs. Several elements are constructed to prepare for the training or inference session. All of these elements are structured into individual class objects, together making up the EngineConfigurations class that is processed by the engine. The following elements make up the EngineConfigurations class and are described in Fig. 4:

1. Dispatcher class: defines the DL action to be performed and the DL technique to be applied.
2. Preprocessing class: defines the routines to apply to the data before they flow into the DL model.
3. TrainData, ValidationData, and TestData classes: contain path locations where the images and annotations will be loaded from and prepared based on the specifications in the Preprocessing class.
4. LearningRate class: defines the learning rate and learning rate schedule to be used by the optimizer during model training.
5. Optimizer class: defines the optimizer to be used during model training.
6. LossFunction class: defines the loss function to be used during model training.
7. Monitors class: defines metrics to be monitored during a model training session (e.g., accuracy).
8. TrainingOptions class: defines a number of parameters related to a training session (e.g., batch size, number of epochs).
9. Saver class: defines data to be saved during training and their associated path locations (e.g., model checkpoints and the locations where they should be stored).
10. Loader class: defines the path to a trained model to be loaded for inference or a model checkpoint to be loaded to continue training (or retrain a model on a new data set).
11. Layers class: contains lists of layers that define the DL model to be constructed. They include separate lists for serial models, for a GAN generator, and for a GAN discriminator.
12. Callbacks class: defines a set of Keras callbacks used to train a model (e.g., save TensorBoard logs). This class is constructed from the Saver, LearningRate, and TrainingOptions classes.
13. Augmentation class: defines the augmentation operation to apply to the training data.

## 5. Illustrative examples

**Fig. 5.**
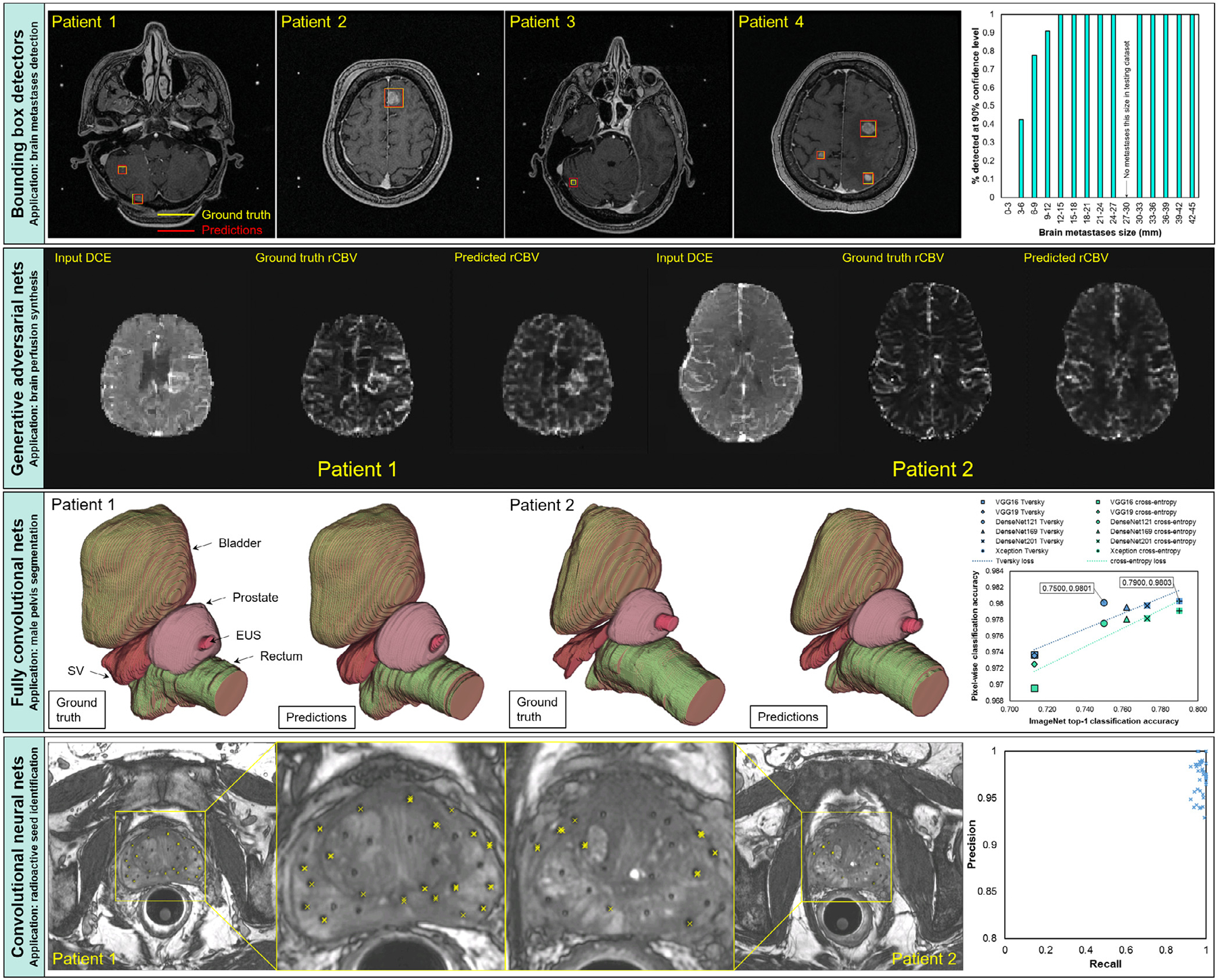
(First band, BBD) (Left) Example detection inferences for four patients with brain metastases. (Right) Detection sensitivity with respect to brain metastasis size at a 90% confidence threshold. (Second band, GAN) A comparison of true and synthetic rCBV maps generated from DCE-MRI. (Third band, FCN) (Left) Inferred radioactive seed locations for two representative patients. (Right) Precision versus recall for 20 test patients. (Fourth band, CNN) (Left) An example set of manual and inferred contours for a prostate cancer patient after brachytherapy seed implantation. (Right).

Some of the primary DL functionalities in DLAE are image classification, image regression, image segmentation, image synthesis, object detection, ablation studies, hyperparameter searches, and DL model deployment. We created one of each of these applications using DLAE to demonstrate some of its core functionalities. Unless otherwise stated, the example applications below were developed using an NVIDIA DGX-1 workstation. Approval from The University of Texas MD Anderson Cancer Center Institutional Review Board was received for all of these applications. Applications 5.1–5.4 are shown in Fig. 5.

### 5.1. Radioactive seed identification for MRI-assisted radiosurgery

Radioactive seed identification after low-dose-rate brachytherapy of prostate cancer is essential for postimplant quality assurance. Currently, a CT and/or MRI is acquired after seed implantation to verify the radioactive seed locations and compute the radiation dose delivered to the prostate and surrounding normal tissues; a medical dosimetrist manually identifies the radioactive seeds. To demonstrate the image classification and regression functionalities of DLAE, it was used to create a sliding-window CNN to automatically perform radioactive seed identification and localization using postimplant prostate MRI [20,21]. The MRI acquisitions were performed with a 3D fully balanced steady-state free precession pulse sequence. Typical scan parameters were: TR/TE/FA = 5.29 ms/2.31 ms/52^◦^, readout bandwidth = 560 Hz/pixel, field of view 15 cm,voxel dimensions = 0.59 × 0.59 × 1.20 mm (interpolated to 0.29 × 0.29 × 1.20 mm), and total scan time of 4–5 min. A sliding-windowalgorithm was written to scan the prostate in three-dimensional subwindows. A chain of CNNs was trained to perform radioactive seed identification and localization tasks. One CNN was trained to classify seed subwindows from background subwindows through cross-entropy minimization. Another CNN was trained to localize the centroids of the radioactive seeds in the seed subwindows through mean-squared-error minimization. The seed centroid locations from the seed subwindows were mapped back to the global image. The CNNs were trained on subwindows extracted from 18 patients. For 20 test patients, the mean ± one standard deviation precision was 96.9% ± 1.9%, and the recall was 98.0% ± 2.1% (Fig. 5). The inference time was about 2 min per patient. The root mean square error of the seed locations was 0.18 mm ± 0.04 mm.

### 5.2. Pelvic anatomy contouring in prostate brachytherapy MRI

The second component of postimplant quality assurance after low-dose-rate brachytherapy is to contour the prostate and its surrounding anatomical structures, including the rectum, seminal vesicles (SV), external urinary sphincter (EUS), and bladder. With knowledge of the organ contours and radioactive seed locations, objectively quantifying the radiation doses delivered to the prostate and these structures using a commercial treatment planning system is possible. Currently, these structures are manually contoured by radiation oncologists. To demonstrate the segmentation and ablation study/hyperparameter searching functionalities of DLAE, we trained six FCNs with different pretrained convolutional encoders and with two objective functions (cross-entropy and Tversky) to perform multiorgan segmentation in postimplant prostate MRI [68,69]. The MRI acquisitions were performed with the same technique as in example application 5.1, and detailed information about the exact acquisition can be found in previous work [70]. The classification accuracy of the pretrained convolutional encoders on ImageNet correlated positively with pixel-wise classification on postimplant prostate brachytherapy MR image segmentation (Tversky loss: y = 0.0949x + 0.9067, R^2^ = 0.8449; crossentropy loss: y = 0.1134x + 0.8908, R^2^ = 0.8586) (Fig. 5). Overall, use of Tversky loss (with *α* = *β* = 0.5 → Dice loss) with an Xception encoder produced greater segmentation accuracy across all the networks than did that of cross-entropy. All model training sessions were performed automatically and in succession using an experiment generator and DLAE. The experiment generator used to perform this study can be found at https://github.com/jeremiahws/dlae/blob/master/fcn_experiment_generator.py.

### 5.3. Brain perfusion synthesis in brain tumor imaging

Dynamic contrast enhanced (DCE)and dynamic susceptibility contrast (DSC)-MRI are perfusion imaging techniques that provide valuable information for diagnosis and evaluation of treatment response in patients with brain cancer [71]. DCE-MRI is best suited for evaluating vessel permeability, whereas DSC-MRI provides robust relative cerebral blood volume (rCBV) imaging. Blood plasma volume can be measured using pharmacokinetic modeling of DCE-MRI. However, the quality is generally inferior to the rCBV obtained from DSC-MRI due to limitations in the sensitivity and temporal resolution of DCE-MRI. Previous studies demonstrated that these two perfusion techniques are complementary and that using both of them provided better diagnostic performance than did using either one alone [72]. However, acquiring both DCE- and DSC-MRI in clinical practice requires two gadolinium-based contrast agent injections, which increase the total dose of the agent. To demonstrate the image-synthesis functionality of DLAE, we trained a conditional adversarial network to map multi-time-point DCE-MRI to rCBV maps computed from DSC-MRI [73] (Fig. 5). The MRI acquisitions were performed on a GE 3T MR750 scanner using an 8-channel brain coil. DCE-MRI was performed by using a 3D spoiled gradient echo sequence with TR/TE/FA = 3.6 ms/1.3 ms/30^◦^, matrix = 256 × 160, 20 slices, slice thickness = 5 mm, 60 phases, and temporal = resolution 5.4 s. DSC-MRI was performed using a gradient-echo EPI sequence with TR/TE/FA = 1500 ms/25 ms/90^◦^, matrix 128 × 128, slice thickness and locations matched with DCE, 60=phases, and temporal resolution = 1.5 s. The tumor:white matter ratio obtained for the synthetic rCBV was significantly correlated with that for the real rCBV obtained via DSC-MRI in the test patient group.

### 5.4. Brain metastases detection in MRI

Presently, brain cancer metastases are manually identified on MR images for stereotactic radiation treatment by radiologists/radiation oncologists, which can be time-consuming. To demonstrate the object-detection functionality of DLAE, a BBD was trained to detect brain cancer metastases. Three-dimensional postcontrast T1-weighted gradient echo images of patients undergoing Gamma Knife radiosurgery were acquired. The MRI acquisitions were performed with a 3D spoiled gradient echo sequence. Typical scan parameters were: TR/TE/FA 6.9 ms/2.6 ms/12^◦^, NEX = 2, matrix size = 256 × 256, FOV 24 × 24 voxel size 0.94 × 0.94 × 1.00 mm. The metastases were manually identified by a neuroradiologist, and bounding boxes were constructed around each metastasis. We constructed a multibox SSD with 16 convolutional layers and predictor layers at six resolution scales. Inputs to the network were axial slices of the MR images, and the outputs were the bounding box coordinates and associated confidences of the classifier. An average sensitivity rate of 73.5% and FP/TP ratio of 15.8 were achieved across all sizes of brain metastases and all confidence thresholds. For metastases at least 6 mm in size, the average sensitivity was 94.5%, and the FP/TP ratio was 7.9 across all confidence thresholds. Overall, the SSD detected almost all brain metastases 6 mm or larger with greater than 90% confidence and nearly half of the metastases smaller than 6 mm with low specificity (Fig. 5).

### 5.5. DL model integration into clinical workflows

**Fig. 6.**
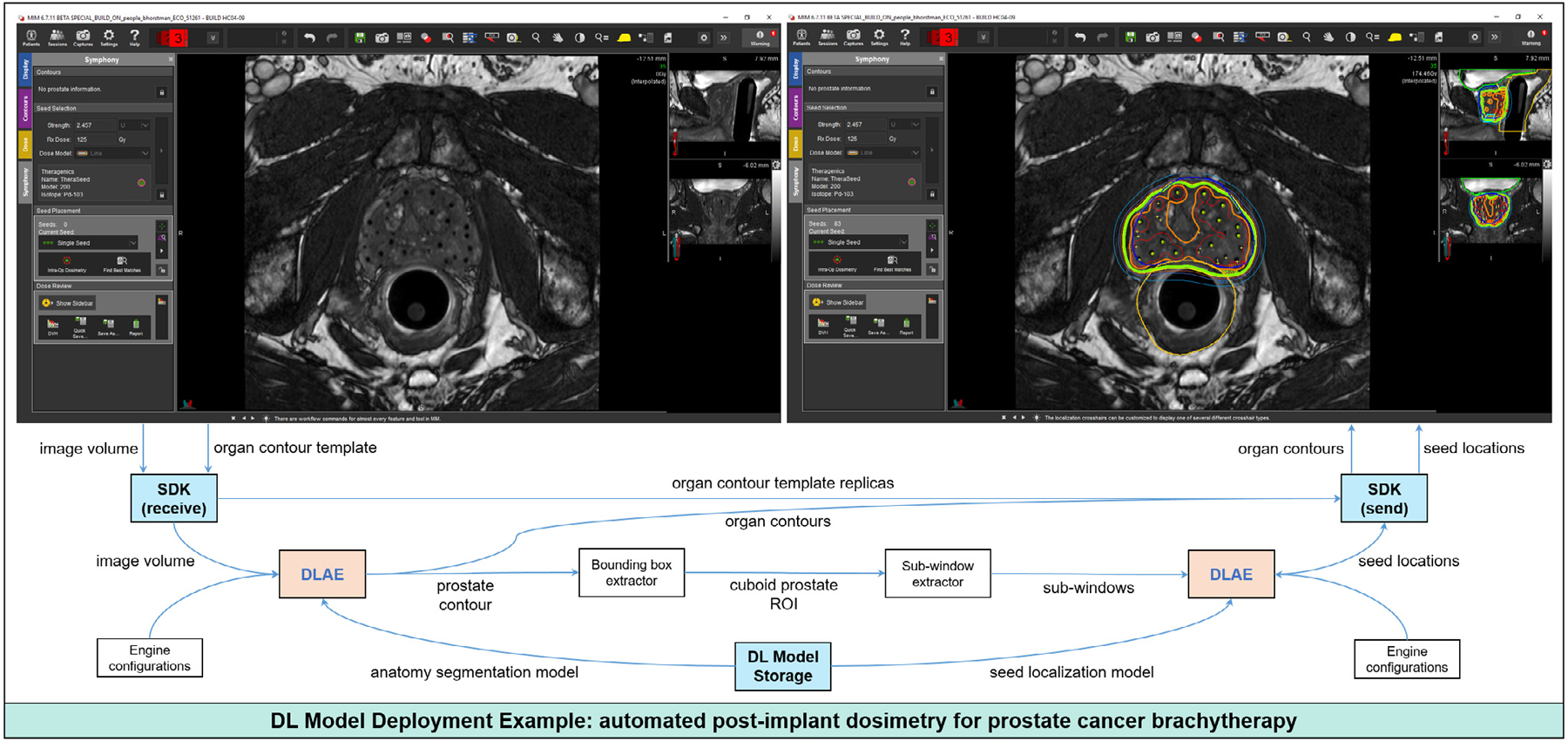
Example integration of trained DL models into a commercial software package with DLAE. An organ contour template and patient images were sent from this program to a MATLAB-based software development kit (SDK). In the SDK, DLAE received the patient images and imported a model trained to segment the prostate, rectum, bladder, SV, and EUS. DLAE then inferred semantic segmentation masks of these five organs. The segmentation masks were stored in five replicas of the organ contour template. The prostate segmentation mask was used to define a cuboid region of interest (ROI) around the prostate for the sliding-window CNN seed identification model. Subwindows were extracted from the cuboid ROI. DLAE then loaded the seed localization model and made inferences of the seed locations within the prostate. The organ contours and seed locations were sent back to MIM using the SDK.

Although developing new DL algorithms in medical imaging for research purposes is important, one ultimate end goal is to make use of the algorithms in a clinical setting. To demonstrate the DL model deployment functionality of DLAE, the sliding-window CNN for radioactive seed identification and the FCN for anatomy segmentation were integrated in a commercial software package used at our institution (MIM Software) (Fig. 6) [74].

## 6. Impact and discussion

DL has become increasingly popular in medical imaging research, and a variety of new medical imaging applications using DL techniques have become possible due to advances in the computer vision field. We present herein a comprehensive software package that encapsulates several of the most popular DL architectures reported in the literature, making explicit use of wellmaintained open-source software libraries. With sufficiently curated data sets, DLAE users can explore a variety of DL techniques to develop new applications in medical imaging. We sought to tailor DLAE to applications involving medical images and allow users to train new models, refine models through ablation studies and hyperparameter searches, retrain models across institutions with different data sets, and incorporate trained DL models into clinical workflows within a single software framework.

We created DLAE as a graphical approach to DL model development as opposed to being explicitly programmatic. This has three benefits. First, we abstracted the tasks of building a new DL model to a series of button clicks rather than explicit computer programming. This makes possible building a DL model quickly across a range of DL techniques. Second, because all of its programmatic implementation details are hidden from the user DLAE may help reduce the learning curve for new DL users and lend itself to expanded collaborations with clinical colleagues who may not have much programming experience. Third, prebuilt DL applications can be loaded and modified with a few button clicks. This enables rapid prototyping of DL models with visual feedback to the user.

Configuration files streamline and automate several aspects of a DL workflow, which facilitate rapid prototyping, development, and implementation of DL algorithms in a clinical environment. Collaborations among research groups on a given application are facilitated by sharing configuration files. Collaborators on a project can share configuration files to load, train, or modify and retrain their DL model. Sharing configuration files, which have a consistent format, may easier than maintaining code from multiple collaborators who have their own styles of coding. Moreover, automation of DL model training and inference with configuration files may enable DLAE to serve as a DL engine in larger software frameworks.

New, more powerful DL architectures continue to be developed in the computer vision field, and the performance of DL models continues to increase. A widely known example is the ImageNet challenge, in which a number of new DL architectures were developed in the literature. As such, these developments demonstrated that not all neural networks perform equally, and developers of DL applications should explore the space of DL solutions and how different network architectures and hyperparameters impact the performance of their models so that they can be confident they have created the highest performing models for a given application. Our example with 12 DL models for segmentation in prostate MRI demonstrated that classification accuracy was positively correlated with pixel-wise classification accuracy in MRI when using a transfer learning approach with convolutional encoders pretrained on ImageNet. DLAE makes it possible to rapidly and automatically perform these types of experiments.

Although we did not present any imaging informatics applications, DLAE lends itself to supporting quality control (QC) programs in medical imaging. Routine QC testing of medical imaging systems has produced large archives of annotated QC data across medical institutions. Imaging physicists can develop DL models with DLAE to perform daily clinical tasks, find the best model for a given QC application, and potentially deploy the application in a QC software program. Moreover, a move toward clinical image-based measurements as opposed to phantom-based measurements for medical imaging informatics applications is underway [75–79]. DLAE can serve as a tool to incorporate DL into these efforts to make use of the many benefits of modern DL techniques.

Because many tasks in a DL workflow can be automated with DLAE using configuration files, DLAE can serve as a support application in a much larger clinical software framework. Some imaging physics groups have constructed in-house informatics frameworks that store, organize, and process large quantities of patient images coupled with their medical data [80]. With some additional software engineering, DLAE could be incorporated into these software packages to serve as the engine for DL applications.

Although the example applications presented were all related to MRI, this is not a requirement. Images acquired from the numerous imaging modalities available in medical imaging can be used to develop DL algorithms with DLAE. Additionally, DL techniques have become popular in several imaging domains. Some examples are optical space imaging, microscopy, art, astronomy, and satellite ground imaging. We originally designed DLAE for imaging applications in medical imaging, but other types of imaging domains could benefit from DLAE. With an approprately curated data set containing images and annotations, DLAE can be used to train algorithms to map input images to a desired output annotation.

The present work has some limitations. First, recurrent neural networks, which can incorporate temporal information into CNNs, are not yet implemented in DLAE. These are a planned update for the next rendition of DLAE. A second limitation is that data curation and model evaluation must be done outside of DLAE. Institutions have unique ways of storing data, and directory structures and file formats are inconsistent across users. Requiring a consistent standard for loading data into DLAE (HDF5 format) was more appropriate than having users conform to a specific data directory structure, which could be confusing and problematic.

## 7. Conclusions

We developed DLAE for end-to-end design, development, and deployment of DL models in medical imaging. A variety of new DL applications in medical physics can be developed and deployed using DLAE and existing institutional data sets. DLAE supports custom model building and contains several prebuilt applications that can be easily loaded by the user. Configuration files decouple the GUI from the DL engine, which enables automation of both training and inference sessions without any user input. DLAE can be integrated into clinical workflows to make patientspecific predictions using patients’ medical images to inform medical decisions. Although familiarity with statistical learning theory is still required for its use, DLAE has a coherent design-todeployment architecture for DL applications in medical imaging, requiring substantially less software development expertise than do existing DL approaches.
